# 3D-QSAR Studies on Barbituric Acid Derivatives as Urease Inhibitors and the Effect of Charges on the Quality of a Model

**DOI:** 10.3390/ijms17050657

**Published:** 2016-04-30

**Authors:** Zaheer Ul-Haq, Sajda Ashraf, Abdullah Mohammed Al-Majid, Assem Barakat

**Affiliations:** 1 Dr. Panjwani Center for Molecular Medicine and Drug Research, International Center for Chemical & Biological Sciences, University of Karachi, Karachi 75210, Pakistan; sajda.ashraf@yahoo.com; 2 Department of Chemistry, College of Science, King Saud University, P.O. Box 2455, Riyadh 11451, Saudi Arabia; amajid@ksu.edu.sa; 3 Department of Chemistry, Faculty of Science, Alexandria University, P.O. Box 426-Ibrahimia, Alexandria 21321, Egypt

**Keywords:** 3D-QSAR, CoMFA, CoMSIA, molecular docking, barbituric acid derivatives, urease inhibitor

## Abstract

Urease enzyme (EC 3.5.1.5) has been determined as a virulence factor in pathogenic microorganisms that are accountable for the development of different diseases in humans and animals. In continuance of our earlier study on the helicobacter pylori urease inhibition by barbituric acid derivatives, 3D-QSAR (three dimensional quantitative structural activity relationship) advance studies were performed by Comparative Molecular Field Analysis (CoMFA) and Comparative Molecular Similarity Indices Analysis (CoMSIA) methods. Different partial charges were calculated to examine their consequences on the predictive ability of the developed models. The finest developed model for CoMFA and CoMSIA were achieved by using MMFF94 charges. The developed CoMFA model gives significant results with cross-validation (*q*^2^) value of 0.597 and correlation coefficients (*r*^2^) of 0.897. Moreover, five different fields *i.e.*, steric, electrostatic, and hydrophobic, H-bond acceptor and H-bond donors were used to produce a CoMSIA model, with *q*^2^ and *r*^2^ of 0.602 and 0.98, respectively. The generated models were further validated by using an external test set. Both models display good predictive power with *r*^2^_pred_ ≥ 0.8. The analysis of obtained CoMFA and CoMSIA contour maps provided detailed insight for the promising modification of the barbituric acid derivatives with an enhanced biological activity.

## 1. Introduction

Urease (EC 3.5.1.5) is a metallo-enzyme that catalyzes the hydrolysis of urea into ammonia (NH_3_) and carbon dioxide (CO_2_). Urease is present in a range of plants, fungi, algae, and bacteria [1,2,3,4] and plays a crucial role in the metabolism of nitrogen during plants’ germination process [3,5]. It is involved in a number of pathogenesis, such as hepatic-coma urolithiasis, pyelonephritis, hepatic encephalopathy, pyelonephritis, urinary and ammonia catheter encrustation [6,7]. The main reasons associated with the above mentioned diseases is followed by the induction of *Heliobacter pylori* which enable bacteria to survive at a minimum pH which causes stomach ulcers (gastric and peptic ulcers) [8]. As a consequence, *H. pylori* infection can induce gastritis and may lead to duodenal and gastric ulcers, gastric lymphoma, and gastric adenocarcinoma [9,10]. Gastric cancer [11,12] is the fourth most recurrent cancer and the next most common reason of cancer related deaths in the world [13]. About 50% of global population is susceptible to *H. pylori*. The obvious remedy for the treatment of infections caused by bacteria with antibacterial agents, up until now, often proves to be ineffective [14]. Consequently, urease is identified as an emerging target for the treatment of bacterial infections related to humans, animals, as well as in cultivation and agricultural science.

Ureases contribute to a basic trimeric array with I, II, or III subunits to fuse making hexameric or dodecameric structure. The active site of this enzyme have two Ni^2+^ ions distant from each other by 3.5 to 3.7 Å, associated by a hydroxide ion and oxygen atoms of a lysine carbamate residue [9,15]. Fungus and plant ureases show only one polypeptide chain, whereas bacteria contain two or three dissimilar subunits (a, b, and c) [4,16]. The integration of Ni^2+^ in enzyme structure is facilitated by accessory proteins, thought to be specific chaperones for urease [17].

The increase of pH in medium by the NH_3_ accumulation is a urease peculiarity of remarkable medical importance [9]. Urinary or gastrointestinal diseases by ureolytic bacteria may lead to complications in human and animal health, consisting of pyelonephritis, kidney stone formation, hepatic encephalopathy and, eventually, hepatic coma [9,15]. Thus, main public health issues are related with *H. pylori*, Gram-negative bacteria that have the ability to survive in an acidic environment as that of the stomach pH. As a result, infection cause by *H. pylori* can provoke gastric inflammation and also increase the risk for the progression of duodenal and gastric ulcers, gastric lymphoma, and gastric adenocarcinoma [9,10]. About half of overall population is sensitive to *H. pylori.* This species of bacteria can survive in stomach of infected individuals for their whole life without causing disease symptoms.

The high prevalence rate of *H. pylori* in the world population specifies that such microbes have developed resistance mechanisms against host defenses [10]. Urease enzyme is the main virulence factor, present in the cytoplasm of *H. pylori* bacteria [18]. It is stated that cytosolic urease release by the lyses of pathogen bacterial cells that attach to the intact bacterial cell surface and cause the hydrolysis of urea. Consequently, NH_3_ formation increases the medium pH, creating a suitable environment for the survival of *H. pylori* [18,19].

During the previous 20 years, the suggested first-line treatment for the eradication of *H. pylori* comprised of the combination of the anti-infection agents clarithromycin and amoxicillin with omeprazole (a proton pump cell inhibitor). Then again, the increment of *H. pylori* imperviousness or resistance to these anti-infection agents (especially to clarithromycin) made this treatment a non-appealing alternative [13,20,21]. Certainly, other treatment methods have developed to battle *H. pylori* disease, which incorporate the utilization of bismuth salts joined with a proton pump cell inhibitor or the combination of different classes of anti-infection agents, such as aminopenicillins, fluoroquinolones, tetracycline, and so on [13,21].

Computer-aided drug design (CADD) approaches have now been regularly used in the designing and identification of new inhibitors in a time- and cost-effective manner [22]. For the last twenty years, ligand- and structure-based drug design strategy has been effectively utilized for the compound’s optimization. In this regard, the 3D-QSAR technique is extensively utilized to develop and predict the bio-activities of novel compounds. Comparative Molecular Similarity Indices Analysis (CoMSIA) and Comparative Molecular Field Analysis (CoMFA) are versatile and powerful 3D-QSAR techniques to build a model for a specified compounds set in drug design and other linked applications [23,24,25,26,27,28,29].

In CoMFA, the conformation selection and molecules alignment are so significant that they influence the quality of the built model. Therefore, with the objective of achieving the opportunity of building a reliable CoMFA model, molecular docking was performed as an alignment tool.

For this purpose, a dataset of 44 compounds were taken from our recently published paper [24]. As different partial charges are applied to compute the CoMFA electrostatic fields, these partial charges can influence the CoMFA model prediction ability. Therefore, we were also concerned to observe different partial charges effect on the accuracy of developed CoMFA model. In this paradigm six different partial charges AM1, AM1BCC, Gasteiger–Marsilli, MMFF94, and Gasteiger–Huckle, charges were evaluated in CoMFA modeling. The model with MMFF94 partial charges produces the best CoMFA model. Consequently, a CoMSIA model was generated by using similar charges. This study has given satisfactory 3D-QSAR results comprising the CoMFA model (*q*^2^ = 0.597; *r*^2^ = 0.897; *r*^2^_Pred_ = 0.818) and CoMSIA model (*q*^2^ = 0.602; *r*^2^ = 0.98; *r*^2^_Pred_ = 0.864). The contour plots achieved from the developed 3D-QSAR models proficiently interpreted the SAR (structure activity relationship). Hence, the generated CoMFA and CoMSIA models are vigorous enough for predicting the compounds activities and further can be used to design more active and selective compounds against *H. pylori*.

## 2. Results

**Scheme 1:**
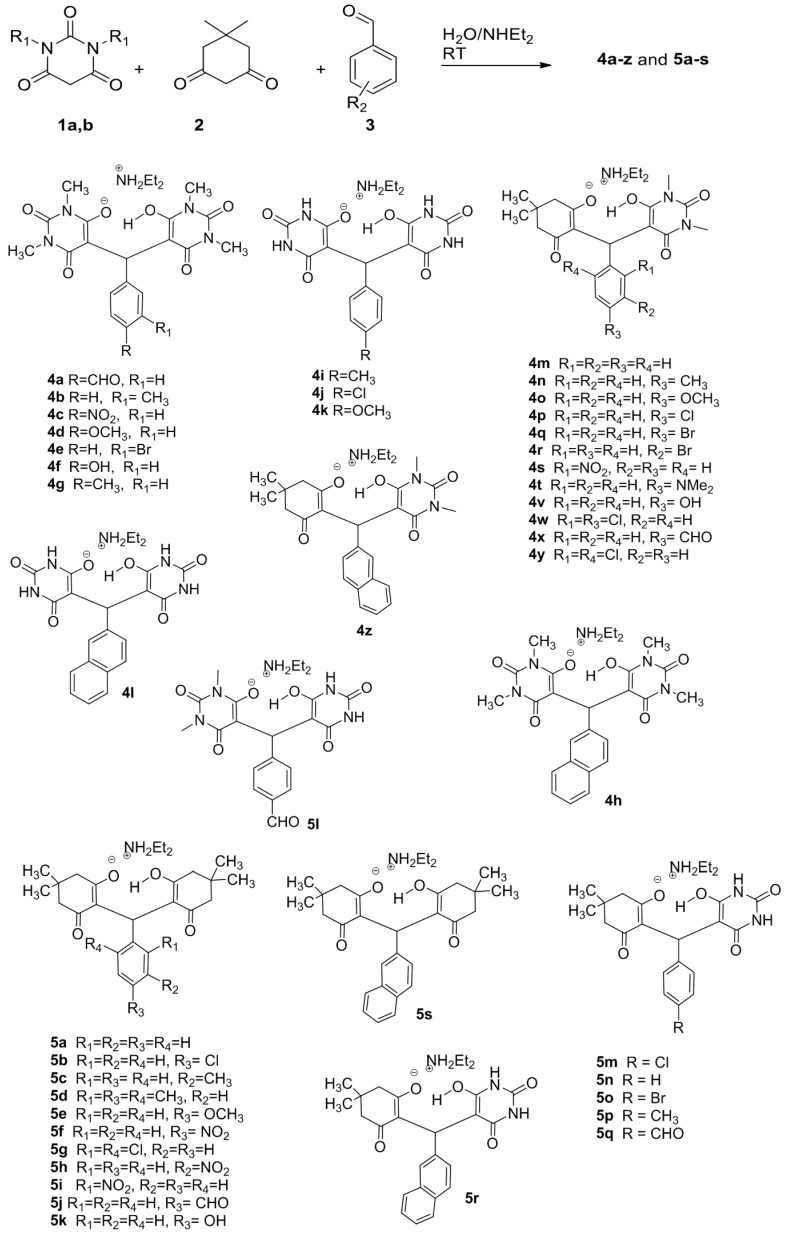
Synthesis of compounds 4a-z and 5a-s.

The studied compounds drawn in Scheme 1 were reported before [24,25,30,31,32]. 3D-QSAR analysis was performed on barbituric acid derivatives. Selections of these derivatives were based on their inhibitory activity against *H. pylori*.

## 3. Discussion

### 3.1. Docking-Based Structural Alignment

Accessibility of *H. pylori* crystal complex structures in the protein data bank (PDB) directed us to carry out a molecular docking study that was intended to examine the molecular interactions of the inhibitors with the target protein. The *H. pylori* crystal complex structure (PDB code: 1E9Y; resolution = 3.0 Å) was taken from PDB. The cognate inhibitor structure (acetohydroxamic acid) was eliminated from protein. All of the compounds were minimized prior to being used in docking experiments.

Partial charges or non-integral charges were calculated by using multiple charge methods from Open Eye and SYBYL 7.3 (Tripos, Inc.: St. Louis, MO, USA, 2007). Moreover, the protonation states of each ligand and ionizable groups were rectified, permitting to the physiological pH by using the Open Eye filter. The molecular docking suite MOE was used for carrying out the docking studies. 

**Figure 1:**
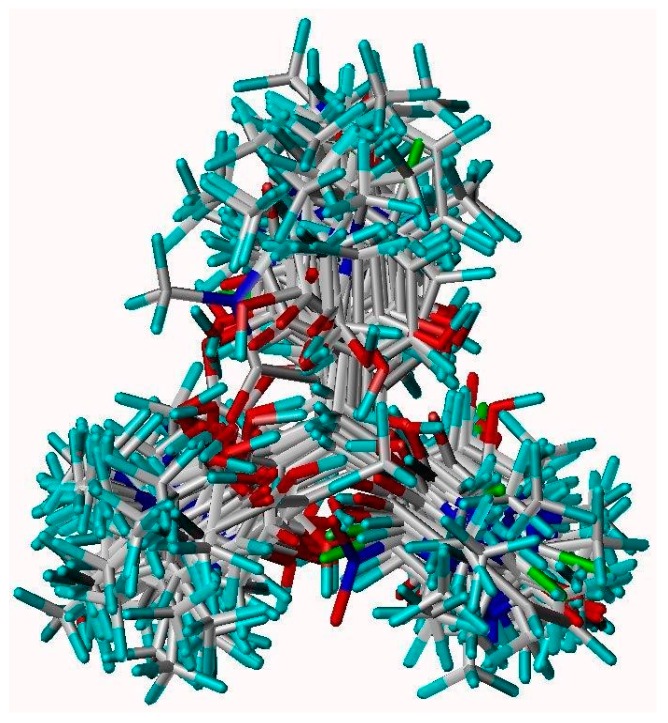
Superimposed view of **1**–**44** compounds by structure-based alignment using the reference ligand of 1E9Y.pdb. Cyan, red, blue, green and white color represent Hydrogen, Oxygen, Nitrogen, Chlorine and Carbon atom, respectively.

The docked conformations of all compounds of particular series of barbituric acid derivatives were depicted in Figure 1. The rest of the compounds were docked by using the aforementioned protocol, and every top-scored confirmation was primarily utilized in advance 3D-QSAR experiments.

### 3.2. Effect of Charges on 3D-QSAR Model

In the present study, different charges methods were used to develop the CoMFA models. It is clearly observed from Table 1 that all the possible partial charges were applied on aligned and docked confirmations of database molecules.

From this study, the best-selected model was extracted with a *q*^2^ value 0.597 and it was obtained by MMFF94 charges with structure based alignment protocol. The PLS study (non-cross-validated) shows a correlation coefficient (*r*^2^) value of 0.897. Moreover, the predictive capabilities of the generated models were assessed by calculating their predictive *r*^2^_pred_ correlation coefficient involving their corresponding test set molecules. The generated CoMFA model with a maximum external predictive ability (*r*^2^_pred_ 0.81) was considered as the best model.

The electrostatic field explained 53.9% of the difference and has a great influence on the developed model as compared to steric field which is about 46.1% of the difference.

Subsequently the development of CoMFA model with the lower standard-errors of estimation (SEE), our generated model satisfied all the conditions of comprising acceptable *q*^2^, *r*^2^, and *r*^2^_pred_ values, so the best model was selected for additional CoMSIA analysis.

**Figure 2:**
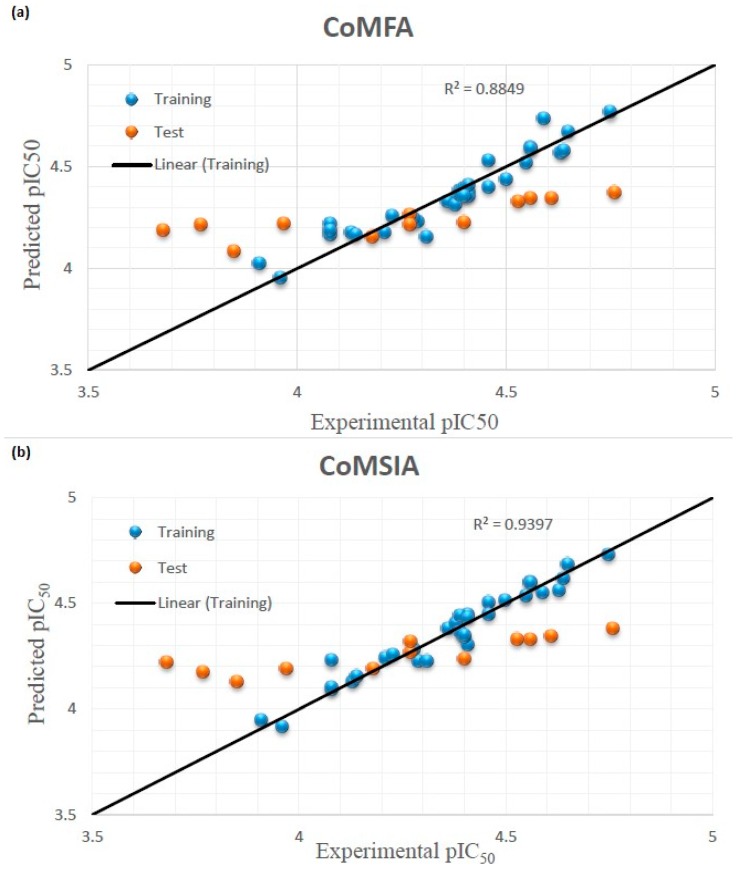
Graphical interpretation of experimental *vs.* predicted pIC_50_ of compounds **1**–**44** developed by CoMFA (**a**) and CoMSIA (**b**) models.

To investigate the numerous field effects on the prediction ability of the models, 23 CoMSIA models were constructed by different field combinations containing electrostatic, steric, hydrophobic, HBD (hydrogen bond donor), and HBA (hydrogen bond acceptor) fields. The developed CoMSIA model with a combination of four fields (steric, electrostatic, hydrophobic, and HBD) descriptors were linked with higher *q*^2^ and *r*^2^ values. The CoMSIA model has a *q*^2^ = 0.602 and an *r*^2^ = 0.98. The model was generated with two components with an *F* value of 209.25 and an SEE value of 0.033. These values recommended that the generated models are consistent and were supposed to have competent predictive ability. The statistical parameters and field contribution associated with CoMSIA models are listed in Table 2. The experimental and predicted bio-activity of the molecules and their difference (residuals) are given in the Table 3, and the graphs are represented in Figure 2.

### 3.3. D-QSAR Contour Maps Analysis

**Figure 3:**
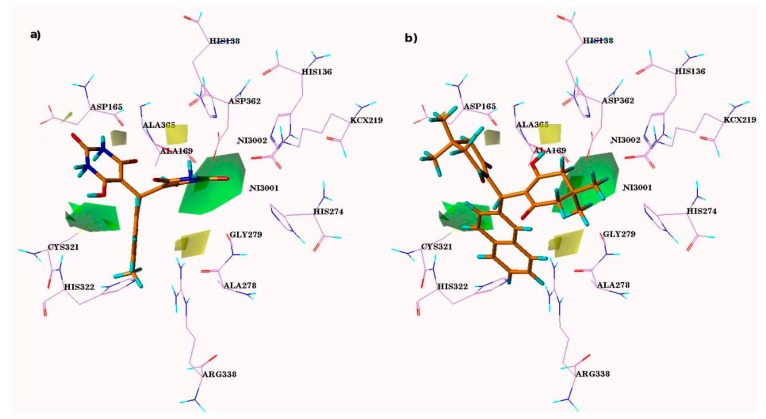

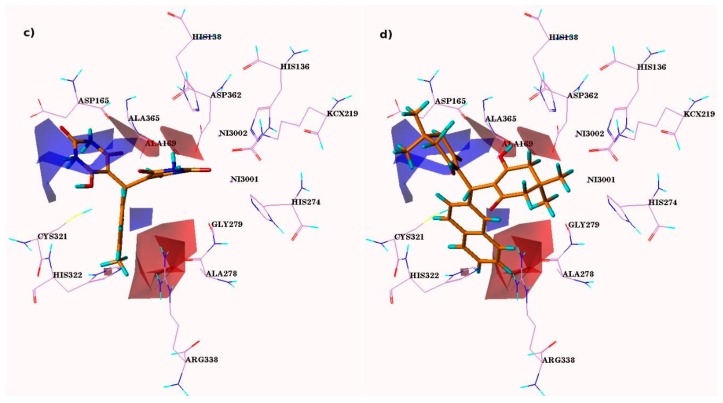
CoMFA steric and electrostatic contour maps. Electronegativity and electropositivity are represented by red and blue contours, while sterically-favored and -disfavored areas are depicted by green and yellow regions, respectively. Panel (**a**) and (**c**) are representative of steric and electrostatic contours of most active comp-4i, while (**b**) and (**d**) are representative of least active comp-**5s**, respectively.

**Figure 4:**
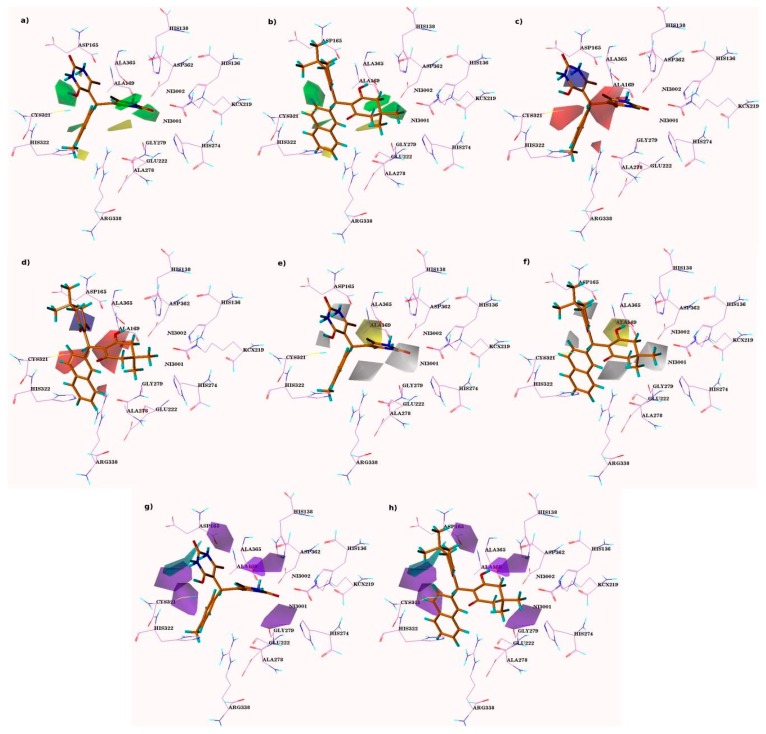
CoMSIA contour maps. Figure (**a**,**c**,**e**,**g**) displayed steric, electrostatic, hydrophobic, as well as donor contour maps of compound 4i claimed as the most active; While, (**b**,**d**,**f**,**h**) are representative of compounds, **5s** claimed as one of the least active compounds within the series.

3D contour plot visualization is one of the striking features in CoMFA and CoMSIA modeling. The contour maps were developed as a product (scalar quantity) of the standard deviations and coefficients, related with every CoMSIA and CoMFA column. These contour surfaces (Figure 3 and Figure 4) described the environment of the protein ligand binding areas.

### 3.4. CoMFA Contour Analysis

In the case of CoMFA, the green contour demonstrates favorable steric interaction, while the yellow contours describe the region where steric interaction is not favored. The area shown by red contours related with the favorable electronegative regions while blue contour favors electropositive region.

The most active compounds **4i** and **5l** were superimposed on the CoMFA contours. Two large blue contour maps were found around the pyrimidine ring of compound **4i** (Figure 3a,c) suggesting the existence of an electron donating group at this position is required to interact with modified lysine KCX219 and Gly279 residue. In the case of least active compounds **4s-4t**, **5f-5g**, **5i**, and **5s** this pyridine ring is replaced by cyclohexenone ring and does not interact with modified lysine KCX219 and Gly279, which significantly lessened the activities of these compounds. Two red contour maps found on the pyrimidine rings near the carbonyl and hydroxyl substituent were representing that the electronegative substitution at this position is suitable for improving its biological activity. These electronegative (EN) moieties interact with NH moiety of catalytic residues (Ala169, Gly279, and Asp362) in protein. Any loss of EN moieties at this position considerably decreases the activity. Another red contour present near the benzene ring of most active compound indicating that any electronegative substitution at this position can enhance the activity. In the least active compounds **4t**, **5g**, and **5i** (Figure 3b,d) carbonyl oxygen is replaced by a different alkyl group, revealing the lesser activities of these compounds.

Two green contours present at the NH group of pyrimidine rings of the compound 4i specify that the bulky substitution are encouraging at these areas whereas the absence of pyrimidine ring considerably decreases the compounds activities (compounds **4t**, **5f-5g**, **5i**, and **5s**).

Correspondingly, another green contour present at the OH moiety of the pyrimidine ring of compound 4i signified that a slight bulky substitution is promising at this point. It revealed that replacement of the carbonyl moiety by the methyl group adjacent to the NH position of the pyrimidine ring of the least active compounds **5f-5g** and **5s**, and the presence of an electron-withdrawing group at the R1 position, were responsible for the lack of activity of **4s**, **5g**, and **5i**. It was investigated that the occurrence of bulky substitution at the meta-position of the benzene ring can further enhance the activity of compounds.

### 3.5. CoMSIA Contour Maps Analysis

The contribution of steric (favored area–85% and disfavored area–15%), and electrostatic (favored area–90% and disfavored area–10%) fields were calculated by CoMFA model. The CoMSIA study is usually in agreement with the CoMFA maps (Figure 3). The sterically-preferable green and non-preferable yellow contour maps occupy similar areas as in CoMFA. In electrostatic maps, negatively-charged regions are also very similar to the CoMFA results. The favorable hydrophobic area (yellow–85%) and non-preferable (white–15%) area, hydrogen bond donor (D)-favored (cyan–85%), and non-preferable (purple–15%) areas, and regions were investigated as the remaining CoMSIA fields.

### 3.6. Steric and Electrostatic CoMSIA Contour Maps

The CoMSIA contours for steric and electrostatic field descriptors were relatively similar with the CoMFA developed models, confirming the reliability of the results. Furthermore, the results of remaining three fields of CoMSIA also enhanced the drug prediction. The CoMSIA contour explains the similar result as CoMFA contour plots. The green contours present near the NH group of the pyrimidine ring of compound 4i showed that bulkier replacements would enhance the activity, whereas the existence of the unfavorable yellow plots near the R position showed that chain elongation or bulkiness can lower the activity of the compound (Figure 4a).

The red color contours in the vicinity of the R group of the benzene ring and the pyrimidine-substituted carbonyl moiety adjacent to NH of compound **4i**, described that the electronegative or electron withdrawing substitution at this location, shows a significant role in the improvement of the inhibitory activity of the ligand. Another red contour found near the hydroxyl group of the pyrimidine ring specifies that this hydroxyl group is significant for the compound’s biological activity.

The occurrence of the blue contour map close to the NH moiety of the pyrimidine ring of compounds **4i** and **5l**, described the significance of the electron-donating moiety at this location to enhance the inhibitory activities of the molecules. This NH moiety mediates hydrogen bond interactions with His322. The CoMSIA contour maps revealed that the absence of aromaticity and the substitutions with the electronegative group at the R position of the phenyl ring reveals that the least active compounds are **4s-4t**, **5f-5g**, **5i**, and **5s**.

### 3.7. CoMSIA Hydrophobic Contour Maps

In the CoMSIA hydrophobic (H) contour map, preferable hydrophobic areas are designated by a yellow surface contour, and the non-preferable hydrophobic regions are presented by white surface maps. At this point, a correlation was made again inside the series of molecules by mapping each compound on the contour map.

The presence of the favored yellow contour map near the hydroxyl of the pyrimidine-substituted ring in compound **4i** specifies the interactions with the active site residue Val169 of *H. pylori*. Substitution of the hydrophobic moiety at this location might improve the biological activity of the molecule (Figure 4e). The disfavored white contour found near the carbonyl moiety of the substituted pyrimidine ring indicates that substitution of the more hydrophilic moiety could enhance the biological activity of molecules **5f-5g** and **5s**. This judgment was consistent with the information that compounds **5f**-**5g**, **5i**, and **5s** were found less active than **4i** and **5l**. Another white contour is observed near the NH of the pyrimidine ring. 

This is the area where Asp168 is present, so the substitution at this region ought to be less crowded.

### 3.8. CoMSIA Hydrogen Bond Donor Contours

In hydrogen bond donor contours, the favorable HBD region was depicted as cyan, while the purple contour recommended unfavorable regions for the HBD field. Again, all molecules were examined by overlaying them on the contour maps and their correlation was observed.

In most active compound **5l** and **4i**, a cyan contour plot representing that region near to NH group of the pyrimidine ring is appropriate as a HBA group (Figure 3g). Two purple contours found near the hydroxyl group of two pyrimidine rings in compound 5l and 4i indicate unfavorable regions for the HBD group positioned in its vicinity. Furthermore, another large purple contour, which is favorable to the hydrogen bond acceptor region, was covering the space near the hydrogen atom located at 3C-position of benzene ring of the same compound.

### 3.9. Building of QSAR Model by MOE

Additionally, to validate our newly developed model, MOE2015 is utilized to generate another 3D-QSAR model. A comparable result obtained from MOE further support the reliability of our developed model. The positive value of n-N (count of nitrogen atom) represents that an increase in the number of N-atoms will also increase the urease inhibitory activity. This judgment was consistent in nitrogen atoms in compounds **4a**–**4l**, which showed higher activity compared to compounds with fewer numbers of nitrogen atom (compound **5f**, **5g**, **5s** *etc.*). The nitro group in compound **5i** is detrimental to biological activity, possibly due to the lack of interaction with the residues Ala169 and Arg338. The contribution of dipole and a_hyd descriptors specify that the urease inhibitory activity is effected by the hydrophobic nature of compounds. The lesser the value of these descriptors, the better is the urease inhibitory activity. The descriptor of polar surface area and Van der Waals surface area also play important roles in urease inhibitory activity. The high values obtained for compound **4e**, **4i**–**4l**, **4r**, and **5n** indicates that to improve the biological activity, large polar surface area for the compounds are recommended.

## 4. Materials and Methods

### 4.1. Dataset Preparation

A set of 44 compounds was taken from our recently published article [24]. Two-dimensional structures of compounds were sketched by Chem-Draw [33] and then converted into three-dimensional structures by MOE2015 (Molecular Operating Environment) software (MOE2015, Chemical Computing Group, Montreal, QC, Canada) [34].

The activities of all barbituric acid derivatives along with its negative log IC_50_ and pIC_50_ values are given in Table 4. The values of pIC_50_ of the dataset set ranging from 3.67 to 4.76 are supposed to contribute to the appropriateness of the training set compounds. Atom typing and stereochemistry of each compound were corrected. Five different charges *i.e.*, AM1, AM1BCC, GH, GM MMFF94 were applied to the dataset. Based on the variety of activity and structure diversity, 44 compounds were divided into training set and test set, containing 36 and 12 compounds, respectively. The molecules of training set are utilized to develop the models, while the test set is used to validate the developed models. The test set compounds evenly covered the chemical diversity and the biological activity range of the database. For structure-based 3D-QSAR, docked conformation was used to align each compound.

### 4.2. Computational Modeling Tools

SYBYL 7.3 package (Tripos, Inc.: St. Louis, MO, USA, 2007) [35] was utilized for performing 3D-QSAR modeling (CoMSIA and CoMFA), on SUSE Linux (Intel^®^Xenon^TM^, Santa Clara, CA, USA) running under an Intel Xeon Quad Core 2.3 GHz. The MOE docking program (MOE2015, Chemical Computing Group, Montreal, QC, Canada) [34] was used to dock the compound’s dataset.

### 4.3. X-ray Crystal Structures of H. pylori

The three-dimensional (3D) X-ray structure of *H. pylori* urease with the resolution 3.0 Å was taken from the protein data bank [36]. Hydrogen atoms were added and all the water molecules were eliminated from the protein. Then docking studies were performed to assess the binding free energy of the small molecule within the macromolecule. For docking purposes, the MOE docking program was used.

### 4.4. Alignment of Compounds Dataset

In the CoMFA and CoMSIA approach, the selection of activity-related conformation and alignment of compounds are two crucial components to build a robust model. Alignment of the individual molecules is one of the vital parameters in 3D-QSAR model development. In Cartesian space, properly-aligned molecules have similar orientation and comparable confirmations. In this study a structure-based (docking) method was used to obtain an active confirmation for the generation of aligned models.

### 4.5. Partial Atomic Charges

With a 3D-QSAR model, variation in partial charges can be affected by the electrostatic contribution in the CoMFA field. Consequently, to evaluate the influence of partial atomic charges on the quality of developed CoMFA models, different charges were applied on the dataset using AM1BCC, AM1, Gasteiger–Huckle, MMFF94, and Gasteiger–Marsilli charges. AM1BCC, AM1, Gasteiger–Marsilli, and MMFF94 charges were calculated using the Open Eye Molcharge program [37] while Gasteiger–Huckle charges were computed with the SYBYL 7.3 (Tripos, Inc.: St. Louis, MO, USA, 2007) [35]. Among them, the model developed by applying MMFF94 charges was considered as the best model. After optimization, compounds were introduced in SYBYL 7.3 for successive 3D-QSAR calculations.

### 4.6. CoMFA and CoMSIA Studies

Comparative molecular field analysis (CoMFA) [38,39] and comparative molecular similarity indices analysis (CoMSIA) [40,41] were performed by using QSAR calculation implemented in SYBYL 7.3 (Tripos, Inc.: St. Louis, MO, USA, 2007) [35]. The CoMFA model derived by two descriptor fields—electrostatic and steric—while the CoMSIA method computes hydrophobic (H) and hydrogen bond acceptor (A)/donor (D) fields, besides electrostatic and steric fields, which are used in CoMFA. These studies were performed for calculating the interaction energy between the aligned compounds and a suitable atom (probe) in a cubic frame of 2.0 Å grid spacing. In each direction, 4.0 Å margin was used so the lattice box has to cover all compounds. Standard parameters of SYBYL7.3 (standard TRIPOS field, energy cutoff 30 kcal/mol and dielectric constant 1/*r*), electrostatic and steric CoMFA fields were examined by a probe atom of sp^3^ positively-charged carbon. In the electrostatic, steric, and hydrophobic fields’ calculation of CoMSIA, a probe atom was used for the Gaussian with distance (dependent) function taking charge +1, hydrophobicity +1, radius 1, and with a default 0.3 value as an attenuation factor.

### 4.7. Partial Least Squares (PLS)/Statistical Analysis

The partial least squares (PLS) approach was utilized for each 3D-QSAR analysis. The correlation between physicochemical properties and CoMFA/CoMSIA molecular fields were achieved by the PLS method. For the present 3D-QSA study, pIC_50_ values were allocated as dependent variables in the PLS examination of obtained models. In this study, leave-one-out (LOO) [38] cross-validated PLS analysis [42] was performed to check cross-validation experiments’ predictive abilities to examine the internal prediction power of the model at every component. The algorithm of PLS with the LOO technique was used to pick the optimal number of components (NOC) and also to evaluate the statistical importance of each model.

The lowest sigma was applied for column filtering up to 2.0 kcal/mol in order to get a better signal to noise (s/n) ratio by eliminating the matrix points with a variation in energy less than the adjusted threshold value. By means of achieved NOC, after the best selected *q*^2^ value carry out the calculation of conventional regression correlation coefficient (*r*^2^).

Validation of the model was done externally by using compounds of the test set that were not involved during model development [39,43]. The predictive ability of 3D-QSAR models was computed by means of the *r*^2^_pred_ value, given by:

*r*^2^_pred_ = (SD − PRESS)/SD

where PRESS is the summation of the square deviations of the actual and predicted activity for every compound in the test set of compounds, SD is the addition of the squared deviations of the experimental and mean bio-activity of the test and training sets, respectively.

### 4.8. Molecular Descriptors

The optimized forty-four structures of urease inhibitors were utilized for calculating 2D and 3D QSAR descriptors. The calculated descriptors included pharmacophore feature descriptors, log P (logarithm of the octanol-water partition coefficient) values, dipole moment, count of nitrogen atoms, surface areas, a hydrophobicity indicator, bond counts, potential energy descriptors, shape, and volume descriptors. These descriptors were empirically computed by an atom-fragment technique executed in the QuaSAR-Descriptor section of MOE.

The possible cross-correlation between independent variables was eliminated by removing descriptor sets that cause cross-correlation values greater than 0.9 (*R* > 0.9). Consequently, 12 conventional QSAR descriptors were designated for QSAR modeling. Thus, the set of final descriptors (depicted in Table 5) were utilized.

### 4.9. Principal Component Analysis (PCA)

**Figure 5:**
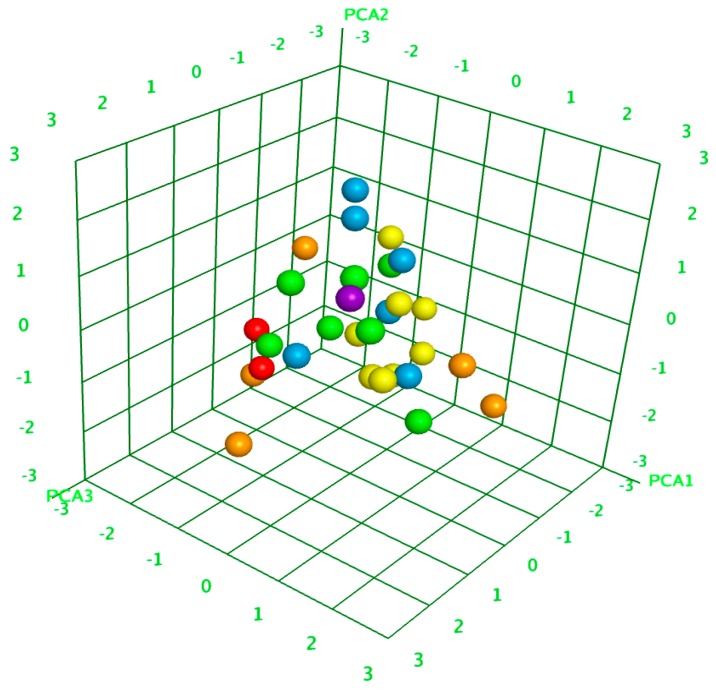
The 3D scatter plot of three selected principle components (PCA1, PCA2, and PCA3), each point corresponds to a molecule and is colored according to its activity.

Principal component analysis (PCA) is a statistical method for data information or elimination of the dimension (number of variables). The principal components are considering as a linear combination of the independent variables [44,45]. The optimized QSAR model contains up to 12 descriptors, which cannot be visualized by 3-D graphics. It is only possible with the top three most appropriate descriptors for presenting the data in 3-D Euclidean space, but it may result in loss in accuracy. To overcome this problem, principal component analysis (PCA) was used, which plots the data components under the QSAR descriptors to 3-D Euclidean space with a minor change. PCA, as executed by the MOE package, first subtracts from every descriptor of each data component, then the mean value of that particular descriptor was calculated. On the basis of this “mean corrected data set”, a covariance matrix was calculated by PCA, where the symbol (i,j) signifies the covariance between the parameters i and j. PCA then calculates the eigenvectors and eigenvalues of the covariance matrix. The principal components of the covariance matrix are eigenvectors that correspond to three highest eigen values. PCA finishes the plotting of the descriptor (original) space into the target 3-D Euclidean space by putting the reference coordinates of the target space as the principal-components of the covariance matrix. The 3D plot of principle components is shown in Figure 5.

### 4.10. Partial Least Squares (PLS) by MOE

MOE was employed to calculate 2D chemical descriptors for each molecule, used for the development of predictive calculations via PLS analysis. Partial least square (PLS) is a method that decreases dimensions in the perspective of regression by means of orthogonal components. PLS merges characteristics of MLR (multiple linear regression) and PCA. The practice gives rise to an optimized equation of a QSAR model with 12 descriptors and the resulting statistical parameters: correlation coefficient (*r*^2^), 0.573; root mean square error, 0.128; cross-validation error, 0.145; and cross-validated *r*^2^, 0.464. The descriptors, including weight (molecular mass of the molecule), TPSA (polar surface area (Å^2^)), vsa_hyd (a pharmacophoric feature that estimates the summation of Van der Waal surfaces of hydrophobic atoms), dipole moment, n_N (count of nitrogen atoms), and zagreb (a directory describing the shape and molecular connectivity of the heavyweight atoms of the molecule) have made major contributions to 3D-QSAR equation development.

## 5. Conclusions

In the current work, two 3D-QSAR models using CoMSIA and CoMFA approaches were developed for a series of recently-published urease inhibitors. The statistical information of both models were found to be satisfactory and comparable. The developed model was noticeably accepted by external and internal prediction and was also supported by visualization of contour maps. The effects of different charges on the developed model were also evaluated. Among these different charges, MMFF94 charge was considered as the best in developing robust and reliable CoMFA and CoMSIA models. Both models’ (CoMSIA and CoMFA) contour plots recommend that electronegative substitution at the *R* position of the benzene ring may play a significant role in enhancing the potency of the compound. Additionally, the substitution of NH of the pyrimidine ring by an electron donating group can also enhance the activity of compounds. The developed model validity was further justified by external prediction. The obtained predicted value of *r*^2^ for CoMFA and CoMSIA models was found to be greater than 0.8. Thus, the developed QSAR models are vigorous enough to direct the design and synthesis of novel and more potent urease inhibitors.

## Figures and Tables

**Table 1:** Partial least square (PLS) statistics summary of CoMFA models in combination with different charges methods.

Models	Charge	*q*^2^	*r*^2^	SEE	SEP	F	NOC	S	E
CoMFA-1	AM1	0.590	0.996	0.014	0.150	124.56	6	0.510	0.490
CoMFA-2	AM1BCC	0.522	0.965	0.042	0.155	184.465	4	0.568	0.432
CoMFA-3	GH^⁄^	0.507	0.970	0.039	0.158	220.940	4	–	–
CoMFA-4	GM^⁄^	0.510	0.935	0.056	0.155	135.194	3	0.494	0.506
CoMFA-5	MMFF94	0.597	0.897	0.071	0.140	81.306	3	0.481	0.519

*q*^2^: cross-validated coefficient (LOO); *r*^2^: non-cross validation correlation coefficient; SEE: standard error of estimate; F: Fisher’s *F*-value; SEP: standard error of prediction; NOC: optimum number of components; S: steric contribution; E: electrostatic contribution; *r*^2^_Pred_: predicted *r*^2^ of test; G–M: Gasteiger–Marsilli and G–H: Gasteiger–Huckel charges.

**Table 2:** Structure-based CoMSIA models along with field contribution in percentage.

Combinations	*q*^2^	*r*^2^	*r*^2^	F	C	SEE	SEP	1%	2%	3%	4%	5%
S + E	0.555	0.808	–	39.401	3	0.097	0.147	21.4	78.6	_	_	_
S + H	0.612	0.954	–	108.02	5	0.049	0.143	15	85	_	_	_
S + D	0.621	0.925	–	51.758	6	0.064	0.144	25.7	74.3	_	_	_
H + D	0.610	0.973	–	148.944	6	0.696	0.146	56.4	43.6	_	_	_
S + E + H	0.572	0.953	–	105.061	5	0.05	0.15	11.9	35.6	52.5	_	_
S + E + D	0.599	0.947	–	93.825	5	0.053	0.145	13.1	40.1	46.8	_	_
S + H + D	0.634	0.978	–	184.087	6	0.035	0.141	08	50.7	40.4	_	_
E + H + D	0.552	0.978	–	183.48	6	0.035	0.156	25.2	42	32.8	_	_
S + E + H + A	0.519	0.925	–	82.96	4	0.062	0.156	8.2	30.2	40.6	21.0	_
S + E + H + D	0.602	0.98	0.84	209.259	6	0.033	0.147	7.3	24	38.5	30.2	_
S + H + D + A	0.509	0.962	–	131.282	5	0.045	0.161	6.5	40.8	31.7	20.9	_
S + E + H + D + A	0.533	0.945	–	115.483	4	0.053	0.154	5.4	20.3	32.3	24.6	17.4

Where: *q*^2^: cross-validation correlation coefficient; *r*^2^: non-cross-validation correlation coefficient; *r*^2^_pred_: prediction of external test set; F Fischer test values; C: optimal number of components; SEE: standard error of estimation; SEP: standard error of prediction; 1%–5%: field contribution of descriptors: S: steric field, E: electrostatic field, H: hydrophobic descriptor, D: hydrogen bond donor field, and A: hydrogen bond acceptor field.

**Table 3:** The actual and predicated pIC_50_ values with their residuals of the training and test set compounds generated by CoMFA-3a and CoMSIA-1a models.

Compounds	pIC_50_	CoMFA		CoMSIA	
	Experimental	Predicted	Residual	Predicted	Residual
**4a**	4.41	4.374	0.03	4.396	0.01
**4b**	4.46	4.399	0.06	4.478	−0.01
4c *	4.26	4.263	0	4.265	0
**4d**	4.5	4.435	0.07	4.489	0.01
**4e**	4.56	4.592	−0.03	4.569	−0.01
4f *	4.26	4.215	0.05	4.318	−0.05
**4g**	4.54	4.517	0.03	4.519	0.03
**4h**	4.39	4.382	0.01	4.389	0.01
**4i**	4.75	4.766	−0.01	4.773	−0.02
**4j**	4.65	4.67	−0.02	4.654	0
**4k**	4.58	4.735	−0.15	4.592	0
**4l**	4.64	4.579	0.07	4.624	0.02
**4m**	4.4	4.355	0.05	4.326	0.08
**4n**	4.38	4.362	0.02	4.36	0.02
**4o**	4.08	4.166	−0.09	4.151	−0.07
**4p**	4.4	4.393	0.01	4.403	0
4q *	4.61	4.344	0.27	4.343	0.27
**4r**	4.56	4.59	−0.03	4.607	−0.05
**4s**	3.96	3.951	0.01	3.974	−0.01
4t *	3.84	4.083	−0.24	4.128	−0.28
**4v**	4.28	4.248	0.03	4.27	0.01
**4w**	4.22	4.256	−0.03	4.246	−0.02
**x**	4.41	4.41	0	4.418	0
**4y**	4.07	4.186	−0.11	4.074	0
**4z**	4.4	4.354	0.05	4.336	0.06
**5a**	4.13	4.175	−0.05	4.115	0.01
5b *	4.53	4.326	0.2	4.331	0.2
**5c**	4.21	4.177	0.03	4.231	−0.02
**5d**	4.29	4.231	0.06	4.263	0.03
5e *	4.4	4.226	0.17	4.238	0.16
5f *	3.97	4.219	−0.25	4.189	−0.22
5g *	3.77	4.214	−0.45	4.175	−0.41
**5h**	4.31	4.156	0.15	4.269	0.04
5i *	3.68	4.129	−0.45	4.322	−0.65
**5j**	4.14	4.164	−0.02	4.154	−0.01
**5k**	4.35	4.325	0.03	4.399	−0.04
5l *	4.76	4.372	0.39	4.372	0.39
5m *	4.18	4.229	−0.05	4.229	−0.05
**5n**	4.62	4.569	0.06	4.634	−0.01
**5o**	4.46	4.529	−0.07	4.485	−0.02
5p *	4.56	4.343	0.22	4.343	0.22
**5q**	4.38	4.313	0.07	4.39	−0.01
**5r**	4.08	4.219	−0.14	4.09	−0.01
**5s**	3.91	4.022	−0.11	3.922	−0.01

* Compounds included in test set.

**Table 4:** *In vitro* activity of urease inhibition of compounds **4a-z** and **5a-s**.

Compounds Name	IC_50_ (μM)	pIC_50_	Compounds Name	IC_50_ (μM)	pIC_50_
**4a**	39.3 ± 0.36	4.41	**4x**	38.5 ± 0.28	4.41
**4b**	34.4 ± 1.57	4.46	**4y**	83.4 ± 1.00	4.07
4c *	54.2 ± 0.47	4.26	**4z**	39.8 ± 1.38	4.4
**4d**	31.6 ± 0.79	4.5	**5a**	74.5 ± 0.88	4.13
**4e**	27.5 ± 0.12	4.56	5b *	29.7 ± 0.67	4.53
4f *	54.2 ± 0.83	4.26	**5c**	61.4 ± 1.12	4.21
**4g**	28.5 ± 0.41	4.54	**5d**	51.3 ± 0.45	4.29
**4h**	40.3 ± 0.32	4.39	5e *	39.8 ± 0.75	4.4
**4i**	17.6 ± 0.23	4.75	5f *	106.4 ± 1.49	3.97
**4j**	22.3 ± 0.73	4.65	5g *	170.7 ± 1.55	3.77
**4k**	25.8 ± 0.23	4.58	**5h**	49.0 ± 0.55	4.31
**4l**	22.7 ± 0.20	4.64	5i *	210.1 ± 0.29	3.68
**4m**	39.3 ± 0.79	4.4	**5j**	72.6 ± 0.59	4.14
**4n**	41.2 ± 0.58	4.38	**5k**	43.8 ± 0.33	4.35
**4o**	83.0 ± 0.66	4.08	5l *	17.2 ± 0.44	4.76
**4p**	39.7 ± 0.70	4.4	5m *	65.9 ± 0.61	4.18
4q *	24.6 ± 0.42	4.61	**5n**	23.7 ± 0.57	4.62
**4r**	27.5 ± 0.19	4.56	**5o**	34.6 ± 0.79	4.46
**4s**	109.7 ± 1.10	3.96	5p *	27.4 ± 0.54	4.56
4t *	142.1 ± 0.64	3.84	**5q**	41.6 ± 0.41	4.38
**4v**	52.2 ± 1.26	4.28	**5r**	82.8 ± 0.72	4.08
**4w**	59.4 ± 0.98	4.22	**5s**	123.2 ± 0.37	3.91

* represents the test set compounds.

**Table 5:** Molecular Descriptors Calculated with MOE.

Code	Description
a_acc	Number of hydrogen bond acceptor atoms
a_don	Number of hydrogen bond donor atoms
a_hyd	Number of hydrophobic atoms.
vsa_acc	Approximation to the sum of VDW surface areas (Å2) of pure hydrogen bond acceptors
Q_PC+PEOE_PC+	Total positive partial charge
Q_PC-PEOE_PC−	Total negative partial charge
i3D	Dipole moment
1K	Kier first shape index
2K	Kier second shape index
3K	Kier third shape index
Weight	Molecular weight (including implicit hydrogens) in atomic mass units with atomic weights
SlogP	Log of the octanol/water partition coefficient.
TPSA	Polar surface area (Å2)
a_nN	Number of nitrogen atoms
